# The potential of patient-based nurse staffing – a queuing theory application in the neonatal intensive care setting

**DOI:** 10.1007/s10729-024-09665-8

**Published:** 2024-01-30

**Authors:** Sandra Sülz, Andreas Fügener, Michael Becker-Peth, Bernhard Roth

**Affiliations:** 1 Erasmus School of Health Policy & Management, Burg. Oudlaan 50, 3062 PA Rotterdam, The Netherlands; 2 Department of Supply Chain Management & Management Science, University of Cologne, Albertus-Magnus Platz, 50923 Cologne, Germany; 3 Rotterdam School of Management, Burg. Oudlaan 50, 3062 PA Rotterdam, The Netherlands; 4 Department of Neonatology and Paediatric Intensive Care, Children’s Hospital, University Hospital Cologne, Kerpener Str. 62, 50937 Cologne, Germany; 5 Department of Business Administration and Health Care Management, University of Cologne, Albertus-Magnus Platz, 50923 Cologne, Germany

**Keywords:** Nursing demand, Flexibility, Quality, Queuing, Staffing, Operations research, Operations management

## Abstract

Faced by a severe shortage of nurses and increasing demand for care, hospitals need to optimally determine their staffing levels. Ideally, nurses should be staffed to those shifts where they generate the highest positive value for the quality of healthcare. This paper develops an approach that identifies the incremental benefit of staffing an additional nurse depending on the patient mix. Based on the reasoning that timely fulfillment of care demand is essential for the healthcare process and its quality in the critical care setting, we propose to measure the incremental benefit of staffing an additional nurse through reductions in time until care arrives (TUCA). We determine TUCA by relying on queuing theory and parametrize the model with real data collected through an observational study. The study indicates that using the TUCA concept and applying queuing theory at the care event level has the potential to improve quality of care for a given nurse capacity by efficiently trading situations of high versus low workload.

**Supplementary Information:**

The online version contains supplementary material available at 10.1007/s10729-024-09665-8.

## Highlights

- The paper focuses on short-term staffing decisions influenced by short-term variations in patient mix and illustrates and approach when and where staffing an additional nurse generates the highest value.
- We apply queuing theory in a neonatal intensive care unit and collect data on care event level to parametrize the queuing model.
- The case study discusses the implications of different staffing levels and reflects on challenges and implications for practice.

## Introduction

Developed countries are facing a severe shortage of nurses. Staffing vacancies remain unfilled despite a growing trend to “shop” other countries to lure nurses from abroad [1, 2]. Meanwhile, health policy regulations have imposed further mandatory staffing levels and are gaining dominance [3–7]. While these health policy regulations are implemented with the best intentions, healthcare providers are struggling to comply with these requirements amid a nurse shortage. Healthcare providers thus need to staff these scarce personnel resources optimally, and the specified number of nurses is crucial here. If providers had an unlimited supply of nurses, they could match nurse supply with nursing demand to fulfill most patient care.

Yet, supply–demand imbalance prevails. Even when healthcare providers enlist a flex pool of nurses to buffer short-term demand surges or nurse supply shortages, this flex-pool method still has its limits. Staffing nurses from a flex pool involves a trade-off: when nurses are staffed in one clinical area, these nurses cannot serve alternative clinical areas. Similar considerations also complicate staffing decisions *within* a clinical department: with fixed roster of department nurses, more nurses staffed to work the early shift means fewer assigned the late shift. Staffing an individual nurse can thus not only be expressed as direct personnel costs, but also as the opportunity cost of not being able to staff the nurse elsewhere.

From a planning and staffing perspective, nurses should be staffed to shifts where they generate the highest positive value for the quality of healthcare. This requires identifying the incremental benefit of staffing an additional nurse. Relying on an innovative application of queuing theory, this paper shows how this quantification can be done. We conduct a case study in a neonatal intensive care unit (NICU), show how to utilize queuing theory to improve staffing decisions, discuss the potential of such an approach, and reflect on practical implications and challenges thereof.

Queuing theory has enjoyed notable application in the operations community where arrivals and departures of patients are typically modeled as stochastic processes (see, for instance, the reviews by Bai et al., 2018 [8] or Lakshmi and Sivakumar, 2013 [9]). Armony et al. (2017), for instance, analyze conditions where step-down units as intermediates add value if set up between critical care units and general wards. These authors relied on queuing theory to model arrival and discharge at the individual patient level to inform strategic capacity decisions [10]. In the context of nurse staffing, early applications of queuing theory are provided by the work of De Véricourt and Jennings (2011) who model excessive delays experienced by patients [11] and Yankovic and Green (2011) who model the interrelations between the demand for inpatient beds and nurses [12]. The case study presented in this paper complements this work by focusing on short-term staffing decisions influenced by short-term variations in patient mix. We analyze a more operational scheme and apply queuing theory at the level of individual-care events. This modelling choice is important in the context of critical care where a delayed fulfilment of an individual-care event may prove fatal.

Real-world settings usually involve staffing levels fixed over time based on the *average* workload. Yet, such models usually do not incorporate *dynamic* aspects, e.g., patient-based differences over time. This is problematic since a mismatch between nurse capacity and patient needs is inevitable, either leading to (opportunity) costs from idle nurse capacity in cases of overstaffing or to high workload levels in cases of understaffing. Excessive workload and understaffing not only negatively affect patient outcomes [13–19]. They also correlate with overloaded nurses, which incurs work dissatisfaction, burn-out [14] and nurse turnover [20, 21]. Therefore, proper alignment of nurse capacity and patient demand potentially can also boost the quality of care delivery indirectly through better working conditions.

For the critical care setting in general and the NICU specifically, timely fulfillment of care demand is essential for the healthcare process and its quality. We therefore propose to measure the incremental benefit of staffing an additional nurse through reductions in *time until care arrives* (TUCA). We assume that patient-generated care events can be modeled as a stochastic process, set up a queuing model, and parametrize this with real data. Our case study specifically intends to capture information about potential systematic temporal variation in nursing requirements otherwise obscured in average workload statistics frequently used in patient classification systems [20, 22]. As such, data needs to reflect the inherent demand variation both among patients and during patient trajectory [23] but this information is not directly available from health information systems. To parametrize the queuing model with real data, we therefore collected this data manually through an observational study. Based on this we quantify the potential of flexibly responding to variations in the expected care demand and analyze counterfactual scenarios to establish an efficiency frontier that can inform staffing decisions when nurses are scarce. We close the paper with reflecting on challenges and implications for practice.

## Analyzing the NICU as a queuing system

Assuming that patient-generated care events can be modeled as a stochastic process, we analyze the NICU as a queuing system. In this section we discuss the main model assumptions and briefly list model input and output parameters. We subsequently parametrize the queuing model with real data in Section 3.

### Model input: arrival and service rates

The model relies on basic queuing theory and makes the following assumptions. Each patient issues care-demand events through a random process. The expected number of care events issued during a defined time period per patient ${\text{p}}$ is denoted as ${\uplambda }_{{\text{p}}}$. In queuing theory, this comprises the arrival rate. The average duration of a care event issued by patient ${\text{p}}$ is denoted as ${1/\upmu }_{{\text{p}}}$. Thus, ${\upmu }_{{\text{p}}}$ describes the expected number of care events one nurse could handle during one time period – denoted as service rate in queuing theory.

In this line of reasoning, the NICU consisting of a set of patients ${\text{p}}\in {\text{P}}$ will issue care events at rate $\uplambda$ with an expected duration of ${~}^{1}\!\left/ \!{~}_{\mu }\right.$ using:

1 $$\lambda ={\sum }_{p\in P}{\lambda }_{p}$$

2 $${~}^{1}\!\left/ \!{~}_{\mu }\right.=\frac{{\sum }_{p\in P}\left({~}^{{\lambda }_{p}}\!\left/ \!{~}_{{\mu }_{p}}\right.\right)}{\lambda }.$$

While the arrival rate for the unit simply equals the sum of the arrival rates of all patients, the expected duration of a care event is the expected duration of care events per patient *weighted* by the respective arrival rate [24].

We develop the model based on the assumption that both care-event arrivals and the duration of providing care can be modelled as Markov processes: the number of care events per time period follows a Poisson distribution with stationary arrival rates, and the duration of care events follows an exponential distribution. We further propose an approximation to account for deviations from those assumptions.

While the first assumption (Poisson distribution of occurrences) has been widely favored in many real-world stochastic settings, the second (exponential distribution of durations) has often faced challenge. Exponentially distributed durations are characterized by high variance (coefficient of variation = 1), a heavy proportion of brief durations, and few very long durations – attributes that may not match typical service processes that instead exert (log) normal distributions.

Critical care as provided in the NICU, however, might be one example where exponential durations fit reasonably well. Most care processes are very brief in nature (such as quick checks of routinely monitored cardio-respiratory parameters or respirator settings), while some take a medium level of time (such as blood sampling, administration of drugs, and oral or endotracheal suctioning). Rarely do care events demand extended times (such as assisting endotracheal intubation, chest drainage insertion, or establishing a central venous access). Thus, exponential durations seem apropos for the critical care setting (and we validate this for our empirical setting). Assumed Poisson occurrences and exponential durations feature quite commonly in the research of critical care settings (see, for example, [25–27] or a summary in [8]). Please note, however, that those studies analyzed data at the patient level spotlighting the arrival of patients and their lengths of stay in a critical care unit, whereas our study models occurrence and duration of care events.

As NICUs serve many patients with a variety of care demand, these (arrival) rates and care durations for each patient need to be estimated and aggregated to derive overall system rates (Eqs. 1 and 2). To ensure feasibility, it may be useful to categorize patients into types where rates can be estimated more easily. An easy categorization of patient types needs crafting but results in the following trade-off. The greater the patient-type differentiation, the more uncertainty may be reduced within arrivals and service times, but this complicates the set-up and operation of the resulting policy. Also, patient types must be easily observable before making the staffing decision. In Section 3, we will use respiratory support to group patients when estimating care-arrivals and care-durations in a real NICU setting.

Another common assumption is stationarity of the arrival process. In the NICU care process, we see two main drivers for potential violations of this assumption: First, the clinical situation of newborn infants is affected by external factors such as general NICU care processes and acoustic levels, with the consequences of different levels of need for care at day and night [28]. Second, ward policies may change within the shifts, for example, certain care events (such as cleaning activities) are performed more often during day shifts than during night shifts. In this paper, we approach this issue by differentiating between shifts, and only assume stationarity of arrivals within each shift.

### Model output: time until care arrives

Assuming Poisson arrivals and exponentially distributed durations, the system is equivalent to an M/M/c system, where ${\text{c}}$ is the number of nurses working in the unit [24]. Based on the arrival rate of care events $\uplambda$, the average event duration ${~}^{1}\!\left/ \!{~}_{\mu }\right.$, and the number of staffed nurses ${\text{c}}$, we calculate the probability that a care event cannot be treated immediately (where all nurses are busy) by applying Erlang’s C-Formula,

3 $${\text{C}}\left({\text{c}},\frac{\uplambda }{\upmu }\right)={\left[1+\left(1-\frac{\uplambda }{\mathrm{c\mu }}\right)\left(\frac{c!}{{\left(\frac{\lambda }{\mu }\right)}^{c}}\right){\sum }_{k=0}^{c-1}\frac{{\left(\frac{\lambda }{\mu }\right)}^{k}}{k!}\right]}^{-1}.$$

From this, we derive the expected waiting time of a care event, i.e., the expected *time until care arrives* (TUCA) as:

4 $${\text{TUCA}}\left(c, \lambda , \mu \right)=\frac{{\text{C}}\left({\text{c}},\frac{\uplambda }{\upmu }\right)}{c\mu -\lambda }.$$

Using this equation, we calculate the TUCA for each arrival rate and expected duration of care events, and the tally of nurses staffed (i.e., for any potential situation). As in other queuing models, waiting time convexly decreases in ${\text{c}}$ (i.e., number of nurses – see Dyer and Proll 1977 for formal proof [29]) while convexly increasing in both $\uplambda$ (i.e., arrival rate of care events) and ${~}^{1}\!\left/ \!{~}_{\mu }\right.$ (i.e., duration of care events). Taking $\uplambda$ and ${~}^{1}\!\left/ \!{~}_{\mu }\right.$ as given, we determine the TUCA for each staffing situation ${\text{c}}$, thus forming the basis for the approach to evaluate potential staffing policies.

One of the benefits of the Markov assumption is that it allows for an exact calculation of our performance measure (TUCA). However, in some situations, arrival and service processes might not exert these properties. For these situations, we propose a simple adaption of the model based on the Kingman Approximation Equation [30] (an extension of the original Kingman’s Formula stated in [31]) that allows for general distributions of arrival and service processes:

5 $$E\left(TUCA\right)=\frac{{cv}_\lambda^2+{cv}_\mu^2}2\cdot\frac{\rho^{\sqrt{2\left(c+1\right)}-1}}{c(1-\rho)}\cdot\frac1\mu$$

Thus, higher (lower) coefficients of variation for the arrival and service process lead to higher (lower) expected values of TUCA. According to Weber (1980), marginal analysis as described in the following section is efficient for G/GI/c systems as well [32]. Therefore, the approach to evaluate potentially staffing policies may be applied equally well based on the approximated values of TUCA, even though this generalized model provides only an approximation.

We acknowledge that TUCA is an aggregated performance indicator that does not differentiate between critical and non-critical care events. In the NICU setting, some care needs will induce prioritization – an urgent need for respiratory support will be handled immediately, whereas a less urgent event will be handled afterwards. A queuing model that differentiated between critical and non-critical care needs would show that decreasing staffing levels would barely affect the waiting times for critical care events but dramatically affect the waiting times for non-critical care events. As an aggregated performance indicator, higher levels of TUCA indicate strongly increasing waiting times for non-critical care events with negative effects on quality of care, and to a lesser extend an increasing risk of having to wait for critical care events. Thus, we treat TUCA as a single-dimensional, easy to understand measure of quality of care within the NICU setting.

### Evaluating staffing policies through TUCA reduction

As described above, nurses are a scarce resource, and we must staff carefully to achieve the best level of care for the given capacity. This helps balancing needs between departments or within departments over time. We propose an efficient procedure to determine staffing levels for given patient mix scenarios, where efficient means that there is no alternative staffing policy with the same number of required nurses and a lower average value of TUCA over all possible patient mix scenarios. Such an evaluation assumes that the arrival rates are known in advance and that the number of nurses can be adapted accordingly. Obviously, these are strong requirements. In practice, we will not face full information and we would have to deal with imperfect forecasts since the critical care setting is characterized by demand uncertainty. However, the arrival of some patients is known some time in advance, e.g. if complex procedures are planned that require observation at the critical care unit afterwards. And in the neonatal care setting it holds that the most vulnerable patients have a reasonably high length of stay at the unit – extremely low birth weight and very-low birth weight infants have an average length of stay of more than 5 weeks (SD: 29 days) [33]. The patients and their health trajectories are thus (at least partially) known to the unit. The second assumption relates to flexible adaptations of staffing levels and while this is challenging, it is not infeasible. Float pool of nurses and on-call schemes are means to address this and we will come back to this while discussing practical implications.

To attain efficient allocation of nurses, hospital management should define the value TUCA reduction per additional nurse staffed. Since ${\text{TUCA}}\left(c, \lambda , \mu \right)$ is non-increasing and convex in ${\text{c}}$, allocation of resources based on marginal values [34] adds the next resource to the system where it provides the most additional value to achieve the highest total reduction in TUCA per given resource consumption. Given that the number of nurses $c$ is an integer, we can define the reduction of TUCA per additional nurse directly:

6 $$\Delta TUCA\left(c, \lambda , \mu \right)=TUCA\left(c, \lambda , \mu \right)-TUCA\left(c+1, \lambda , \mu \right)$$

This measure can determine the marginal benefit of an added nurse for a given setting. In other words, if the reduction in TUCA, i.e. $\Delta TUCA\left(c, \lambda , \mu \right)$, is higher than a pre-defined threshold $\Delta {{\text{TUCA}}}^{min}$, adding a nurse is worthwhile. The approach we propose allocates resources based on marginal values, i.e. it adds the next resource to the system where it provides the most additional value to achieve the highest total reduction in TUCA per given resource consumption. This is not the same as minimizing the maximum wait time. For instance: If adding one nurse could reduce TUCA from 1.0 to 0.9 in situation 1 and could reduce TUCA from 0.9 to 0.4 in situation 2, the approach proposed in the paper allocates the additional nurse to situation 2 (leaving the maximum wait time at 1.0). But since ${\text{TUCA}}\left(c, \lambda , \mu \right)$ is non-increasing and convex in *c*, both approaches are likely to result in comparable staffing solutions for situations that do not differ substantially in demand. Note that this approach requires a $\Delta {{\text{TUCA}}}^{min }$ threshold that states until which reduction in TUCA an additional nurse should be staffed. Another practical approach (given scarce resources) would be to allocate nurses to situations until the total number of nurses staffed caps out. Such an approach adds a nurse to the situation with the highest $\mathrm{\Delta TUC}{{\text{A}}}_{{\text{s}}}$ until the expected number of nurses (weighted for situation likelihoods) reaches the target capacity.

## Parametrizing the model with real data—the empirical case

### Study setting

The study setting is a pure NICU within a tertiary perinatal center in Germany that provides care for preterm and sick newborn infants. This NICU is an open-bay station using room dividers spaced approximately three meters apart where all infant beds are centrally monitored. Regular capacity of the NICU is 11 beds, and up to two additional beds may be provided when needed. The NICU uses fixed staffing policies with the number of nurses staffed depending on the shift time. Early shifts run from 6:30 am to 2:30 pm with NICU staffing of five nurses; late shifts run from 1:30 pm to 9:30 pm with five nurses staffed, and four nurses attend the night shift from 9:00 pm to 7:00 am. The overall nursing staff of our study unit comprised 52 nurses (equivalent to 31.5 FTE) – all qualified in line with Neonatal Nurse Practitioners (NNPs) and subject to joint planning.

### Data collection

As data on nursing care demand is not available within information systems, it had to be collected prospectively within an observational study that had been approved by the ethics review committee and the staff council of the participating institution. Following Milligan et al. (2008) and Pillay et al. (2012) [35, 36], we collected nursing care data through passive observations. These observations were conducted by study nurses with sufficient medical expertise and background knowledge. Using a tablet PC and a self-modified software application, the study nurses assigned time stamps to nurse activities using the following four categories: (1) direct care activities, defined as activities performed while caring for the infant (e.g., feeding, administration of drugs, and documenting the patient’s data); (2) indirect care activities (e.g., cleaning equipment), which were defined as activities that were not directed to an individual infant; (3) administrative activities such as scheduling and central documentation; and (4) other activities, such as personal needs and staff breaks. The taxonomy is based on the original classification used in [35, 36] and only slightly adapted where required in the German context. The complete list is provided in Appendix A1.

Our analysis focuses on direct care because these are time-critical activities that require nurses to respond in a timely manner and because direct care activities are stochastic and can be neither scheduled nor anticipated entirely.

To limit disturbances in the care process and to conform with the data collection requirements posed by the participating institution, we refrained from continuous observation and instead relied on random sampling techniques and randomized observation intervals [35, 36]. We designed observation blocks of three hours, each block containing 12 observation intervals of 10 min each, with 5-min breaks in between (to allow the study nurse to rest). For each workday, we randomly selected one of the three shifts (early, late, night) as well as a random starting point within the shift ensuring that each observation block falls completely into the shift to exclude handover activities. This gave us a random sample of each type of shift as well as random times within each shift.

Each observation block contained multiple observation intervals. For each observation interval, one of the nurses on duty (and who gave consent to participate in our study) was randomly selected for being observed. This design allowed us to observe a representative random sample of nursing workload at the NICU in our partnering hospital. This resulted in observing one nurse at a time, and the recorded tasks represented the tasks performed by an “average” nurse. According to our approved study protocol, we aimed for 80–90 observation days. Since the observations had to be done by study nurses who also had regular nursing duties, we had to give the study nurses the possibility to combine their research and regular nursing activities. Therefore, we generated a sample with potential observation days and randomized starting times (as described above) for which study nurses could register on a first-come-first-served basis. Due to short-term unavailability of study nurses, we ended up having 84 observation days between 06/2015 and 11/2015. Observations took place during each day of the week and started 33.33% of the time in early shifts, 45.24% in late shifts, and 21.43% in night shifts yielding an unbiased sample. On some observation days, study nurses stopped the observations before the three-hour block was reached due to sickness, being called off from their research activities, or technical failure of the equipment. In some instances, the observation of individual nurses was not permitted because the nurse did not provide consent, or the nurse provided palliative care and the observation was not permitted for ethical reasons. For these reasons, the total observation time amounted to 155 h. During the study period, the average monthly demand was almost identical to the average monthly demand of the year before. Therefore, we are confident that for the study NICU, the choice of the study period did not undermine its generalizability beyond the study period.

### Patient-based care demand and durations

To parameterize the model, we need the NICU’s arrival rate and the expected duration of care events (Eqs. 1 and 2). Care events vary between and within patients. Ideally, we would enlist patient-specific, time-varying parameters were this not practically infeasible. To sidestep time- and patient-specific rates, we require a patient indicator easily observable *ex ante* that well discriminates varying levels of care intensity. From a medical perspective, patient risk and severity indicators might serve best since patients suffering more severe conditions demand greater nurse attention and may trigger more and/or longer care events that correlate with more time providing direct care.

Within the NICU, several patient risk and severity indicators exist, such as birth weight, gestational age [37–39], CRIB score [40], and Apgar score [41]. These factors are routinely collected after birth and offer easy access. Yet, in this case, their static-per-patient nature limits prediction of care intensity variation over time. We thus decided to use respiratory-support type as an indicator of a patient’s level of care intensity, which is dynamic and easily observable [42]. Regarding respiratory support, the highest medical severity is recorded when infants require intubation and mechanical ventilation.

While mechanical ventilation has been applied more often in the past, noninvasive forms are currently replacing their invasive counterparts in medical outcomes [43, 44]. Noninvasive forms, such as nasal continuous positive airway pressure (nCPAP) and high-flow cannulas, support infant respiration with the prerequisite an infant can spontaneously breathe on her own. Therefore, noninvasive forms of respiratory support, in general, reflect a better cardiorespiratory condition and lower severity levels than mechanical ventilation.

In the NICU, a noninvasive respiratory-support type is the first-line therapy. Where intubation is inevitable, the primary goal is expedited extubation and transfer to noninvasive respiratory support. Consequently, we may assume infant respiratory-support type to indicate cardiorespiratory condition that reflects severity differences for both *between* and *within* infants along their health trajectories. This study distinguishes four forms of respiratory support: (1) mechanical ventilation (i.e. invasive with endotracheal intubation or non-invasive), (2) nCPAP (nasal or pharyngeal CPAP), (3) high-flow cannula, and (4) a miscellaneous category describing all infants without respiratory support.

Beyond patient-related factors, structural features inherent in the NICU’s current roster, such as shift types (early, late, and night), likely affect care demand when shifts differ regarding their care procedures. Planned procedures, such as exchange of respiratory support material, routine ultrasound, blood sampling, and scheduled C-sections, occur more often in early shifts. Also, dayshift noise from many health care practitioners and visitors on the ward might irritate infants to the point of triggering care events, i.e. apnea or bradycardia [28]. An empirical analysis demonstrating that a patient’s total care demand differs between shifts and respiratory type can be found in the Appendix A2.1 and A2.2. The case study thus incorporates two aspects relevant for care demand, i.e. shifts (early, late, night) and patient types (four types of respiratory support) and therefore features 3 × 4 different parameters each for arrival rate and expected duration of care events serving as inputs to the model.

**Fig. 1:**
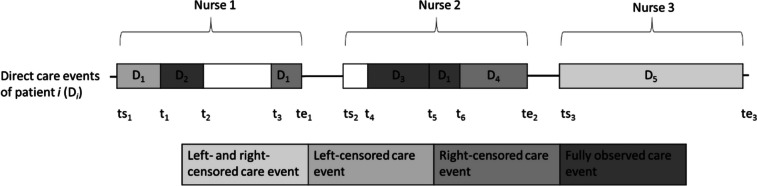
Observation pattern

To derive the parameters for the model we need to look at the observation procedure in detail. The data collection process and the resulting data pattern are illustrated in Fig. 1.

Nurses are observed over predefined intervals. For example, the observation of Nurse 1 starts at ts_1_ and ends at te_1_. At ts_1_, the observer records that Nurse 1 is currently providing direct care for patient D_1_. At t_1_, Nurse 1 switches to patient D_2_ and provides care until t_2_, when she proceeds with indirect care activities. At t_3_, Nurse 1 switches activities again and provides care for patient D_1_. This observation pattern has the consequence that not every care event is observed in its entirety and that some are subject to censoring. For instance, at ts_1_, we observe Nurse 1 providing direct care for patient D_1_; however, the event started before the observation interval, i.e., the event is left-censored. As is the case at te_1_, the observation interval stops, but the care continues, in which case the event is right-censored. Finally, we may have cases where nurses continuously provide care for one patient (D_5_) throughout the entire observation interval, as illustrated in the case of Nurse 3. Here, we observe neither the exact start nor the exact ending of the care event. Although it is challenging to identify the exact duration of care events, this observation pattern allows us to determine the proportion of time that each nurse spends on direct care provision.

To derive the arrival rates of care events, we must calculate the number of care events per minute and patient. To do so, we start by estimating the number of care events within the observed timespan. We require one fixed point to identify individual care events. As a straightforward fixed point, we may consider either the start of the care event or the end of the care event. As described above, we do not observe nurses and the care that they provide in a continuous fashion; hence, we do not observe every care event completely but, for some of them, only the start or the end. Therefore, we consider the arithmetic mean of the observed starts and ends as an unbiased estimate of the number of care events treated by one nurse. To obtain the average number of care events per minute and patient, i.e., the arrival rate, we divide this figure by the total number of observed minutes (leading to the average number of care events for one nurse per minute), multiply by the average number of nurses (leading to the number of care events for the complete NICU), and divide by the average number of patients (leading to the average number of care events per minute and patient).

We illustrate this approach with an example: For infants with nCPAP respiratory support in the early shifts, we observed 131 starts and 117 ends during the observed time interval of 2,847 min (the total observation time in early shifts). To determine the expected number of care events per nurse and minute, we divide 124 (i.e., the arithmetic mean of 131 and 117) by 2,847 min and obtain an average of 0.04 care events from nCPAP infants per minute treated by one nurse. On average, 4.78 nurses were present during our observation periods in early shifts; thus, the complete NICU experienced on average 0.21 care events from nCPAP infants per minute. Given that 7.04 nCPAP infants were present on average, one nCPAP infant leads to 0.03 care events per minute. The left part of Table 1 summarizes the occurrence rate of care events for each patient *i* differentiated by shift and type of respiratory support. We assume that the NICU’s arrival of care events follows a Poisson process with an arrival rate of λ equal to the cumulated arrival rate across all infants ($\lambda =\sum_{p\in P}{\uplambda }_{p})$.

**Table 1:** Input parameters for queuing model

	Average number of care events (arrival rate) per infant per minute					Average duration of care events in minutes			
Respiratory support / Shift	Mechanical	nCPAP	High-flow cannula	Miscellaneous	Mechanical		nCPAP	High-flow cannula	Miscellaneous
Early	0.034	0.030	0.022	0.017	10.76		8.48	7.81	30.11
Late	0.037	0.025	0.017	0.020	8.93		8.54	9.66	15.28
Night	0.038	0.022	0.014	0.029	6.87		6.47	12.25	7.30

Analogous to the arrival rates, we determine the average duration of one care event for each combination of respiratory support and shift. For this purpose, we divide the cumulated observed duration of care events in minutes by the estimated number of care events within the observed timespan. For instance, as stated above, for infants with nCPAP respiratory support in early shifts, we identified 124 care events, encompassing a total timespan of 1,051 min. Thus, the average duration of one care event equals to 8.48 min. We proceed similarly for the other shift-respiratory support type combinations and estimate the average service times of care events and summarize those in the right part of Table 1. We assume that the durations of NICU’s care events follow an exponential distribution with an expected duration of 1/ µ across all infants $({~}^{1}\!\left/ \!{~}_{\mu }\right.=\frac{{\sum }_{p\in P}\left({~}^{{\lambda }_{p}}\!\left/ \!{~}_{{\mu }_{p}}\right.\right)}{\lambda })$. As stated in Section 2, NICU might be one of rare settings where the assumption of exponentially distributed service processes is justified due to a high number of very brief processes. Following the logic of Litvak et al. [25], we assume the number of NICU’s care events per time unit to follow a Poisson distribution, while the duration of NICU’s care events follows an exponential distribution for each patient mix per shift.

As indicated in Table 1, depending on shift type and respiratory support, patient care demand varied 0.8 to 2.3 times per hour (arrival rates of 0.014 to 0.038 care events per minute), and expected care-event duration ran 6.47 to 30.11 min. These parameters serve as basic input parameters for our queuing model that aggregates a patient type’s care-event arrival rates (${\uplambda }_{{\text{p}}}$) and durations (${1/\upmu }_{{\text{p}}}$) on the unit level applying Eqs. 1 and 2. In the following section, we determine the patient mix, i.e., the number of patients per type.

### Patient mix – aggregation across patients and patient distribution

Parameters in Table 1 represent the arrival rate and duration of care events initiated by an individual patient of a specific respiratory type during a specific shift. To analyze the NICU as a queuing system, we need to consider the cumulative arrival (and service) rate across all patients per given patient mix.

The data shows wide variety in utilization and patient mix. Generally, we see potential states of the NICU ranging from 0/0/0/0/13 (0 mechanical, 0 nCPAP, 0 high-flow, 0 miscellaneous, 13 empty beds) to 13/0/0/0/0 (13 beds occupied by mechanically ventilated patients). For each patient mix, we can calculate the NICU’s arrival rate and expected duration via the specified patient-type rates in Table 1. When validating the Markov assumptions for occurrences and durations of care events (see Appendix A3), we conclude that the Markovian features seem to apply for early and late shifts. For night shifts, we rely on the general approximation stated in Section 2.2.

To optimally allocate nurses across multiple shifts and days, we need to estimate the likelihoods that a given patient mix occurs. Using the 84 observation days, we estimate the distribution for number of occupied beds, and the probability for each possible respiratory type. Assuming a multinomial distribution for those input parameters, we can estimate the probability for each possible patient mix. The most frequent situations utilize 11 beds for seven to nine patients under non-invasive respiratory support, and the remaining two to four patients under invasive respiratory support. This distribution allows determination of the overall performance for a certain staffing policy by calculating the average TUCA and number of staffed nurses.

### Analysis of TUCA improvements

Based on the model discussed in Section 2 and the input parameters from Section 3.3 and 3.4, we now assess the impact of different staffing scenarios for the NICU. We engage the following questions for the case study: How much gain in quality of care is achievable using a more flexible staffing policy? Or, alternatively, can the NICU use a more flexible staffing policy that maintains the current quality of care level with fewer nursing hours? Analyzing these two scenarios maps out an efficiency frontier.

The first reference point is the status quo at NICU where a fixed number of nurses attends early shifts (c = 5), late shifts (c = 5), and night shifts (c = 4). For this status quo scenario (5–5-4) and the estimated patient-mix distribution (see prior Section 3.3), the expected time until care arrives (TUCA) across all situations is 1.15 min. We scaled all subsequent outcomes according to this status quo.

We next proceed with alternative staffing levels for fixed staffing scenarios. Here, we use a constant number of nurses staffed per given shift regardless of the unit’s patient mix (shaded grey in Fig. 1). Unsurprisingly, adding resources, i.e., increasing the total nursing hours, improves expected quality of care, while withdrawing resources impairs quality. A 6–5-4 policy, for instance, cuts average TUCA by 41% compared to the status quo, while a 5–4-4 policy increases average TUCA by 61%. Note that we display only the lowest average TUCA for the same number of staffed nurses across shifts (e.g., we do not report plans like 2–5-6 yielding higher average TUCA staffing the same total nurses (13) as 5–4-4).

**Fig. 2:**
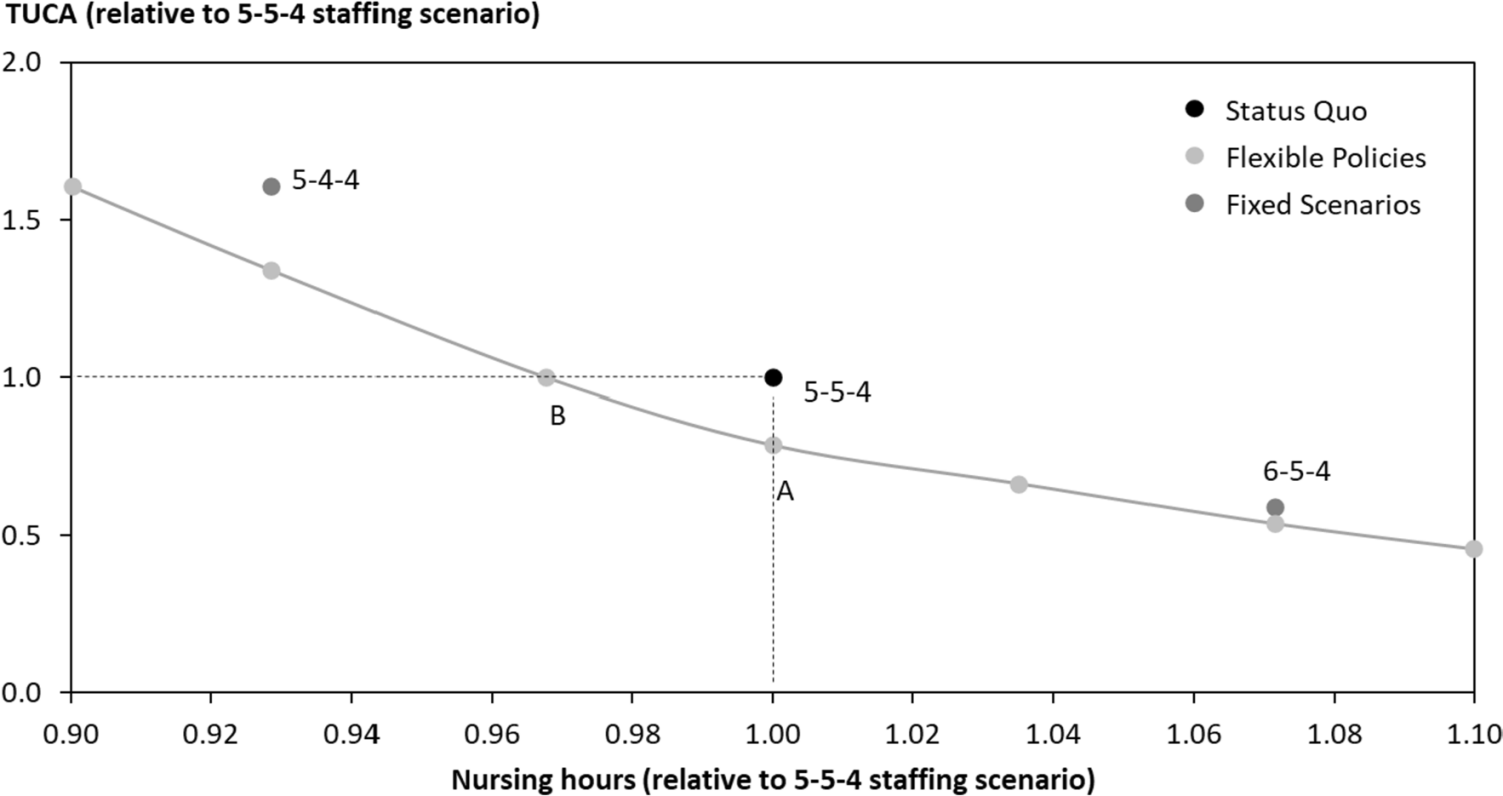
Efficiency frontier

In the flexible staffing approach, the number of staffed nurses depends on the patient mix during that specific shift. We assume that the information on the number of occupied beds and infant respiratory type is known before the shift onset (note that this assumption is relaxed in Section 4) and that the number of nurses can be adapted accordingly. We follow the threshold approach of Section 2.3 where an individual nurse is added to a specific shift as long as the new nurse saves more TUCA than a threshold value. To represent a large range of potential staffing levels, we varied the threshold value from 0.65 min to 3.60 min, and draft an efficiency frontier. Due to the convexity of TUCA in the number of staffed nurses, there is no allocation of nurses that leads to a lower TUCA without increasing the number of staffed nursing hours. The result of the flexible nurse allocation scenarios is depicted in light grey in Fig. 2.

As a result, the flexible staffing allocation can achieve the average status-quo TUCA using 97% of its nursing hours (Point B). In other words, by flexibly adjusting the number of nurses to the patient mix, the unit could have maintained the same quality of care level incurring 97% of the direct nursing costs. Fewer nursing hours that maintain quality of care is interesting not only from a cost-containment view, but also because the current skill shortage impairs hospital staffing. Alternatively, we also see that the unit could have curbed average TUCA by 18% while maintaining its current nurse-hours (see Point A). Note that every point between A and B represents a flexible staffing policy that offers better care at less direct nursing cost, that is, a lower average TUCA *plus* lower expected nursing hours. The reason for the flexible staffing outperformance lies in averting situations of extremely high TUCAs. Of course, avoiding high-TUCA situations by staffing nurses who would have otherwise been squandered in shifts with relatively low TUCAs means that lower TUCAs must rise in response for these less busy situations. Note that the cost savings described above only pertain to the direct nursing costs. Decision-makers wondering whether to implement such a policy might attribute value to a full cost–benefit analysis. In our case, the benefit of decrease of TUCA is easier to measure than the costs, as this is also influenced by the workforce, flexibility in contracts, and possible shift patterns – all of those being outside the scope of our paper. Besides, it would be valuable to identify and quantify all direct and indirect consequences for all affected stakeholders in addition to the implementation and operating activities (equipment, training, maintenance, etc.). We feel that measuring consequences such as satisfaction and well-being of professional staff, a holistic assessment of the patient health status, etc. is worth analyzing in a separate study.

From a clinical perspective, it is worthwhile to consider that the NICU might face limited flexibility in its staffing policy. Notably, within the NICU context, the qualification level of nurses is a constraint, i.e. a critical care unit cannot rely on a general flex pool to supply nurses lacking the required expertise to work in the NICU environment. Let’s say that deviations from the status quo policy (5–5-4) may never exceed ± one nurse. From a practical perspective, adding one nurse could be achieved through calling in staff being on standby whereas withdrawing one nurse could imply that the nurse is assigned to other non-direct care activities (or deployed in a distinct but related clinical area). Adding such a constraint still leads to substantial improvements. Restricting the variability to ± one nurse, average TUCA could still be improved by 17% – a large share of the improvement potential of 18%. Taken together, the results show that applying queuing theory at the care event level has the potential to improve quality of care for a given nurse capacity by efficiently trading situations of high versus low workload.

## Robustness against deviations from the true patient mix

In the main analysis presented in the paper, we assume that the information on the number of occupied beds and infant respiratory type is known before the shift onset and that the number of nurses can be adapted accordingly. In this section we test the robustness of the results when this assumption is relaxed. Specifically, we consider scenarios in which the true patient mix is different than the a priori assumed patient mix upon which the number of nurses is staffed. The true patient mix can deviate for two reasons: i) because the number of patients turns out to be different than the anticipated number or ii) because the respiratory types turn out to be different than the anticipated ones. To test the robustness of the results, we conduct a comparative analysis based on different scenarios in which the true patient mix does not resemble the planned patient mix used for adjusting staffing levels. The baseline scenario is the status quo, i.e. the observed patient mix with fixed staffing levels. We then simulate deviations from the observed patient mix. For each simulated patient mix we derive the NICU’s expected arrival rate, expected service duration, staffing level and TUCA. Each of the simulated scenarios presented below relies on 1,000 repetitions.

### Mis-specified number of patients

**Fig. 3:**
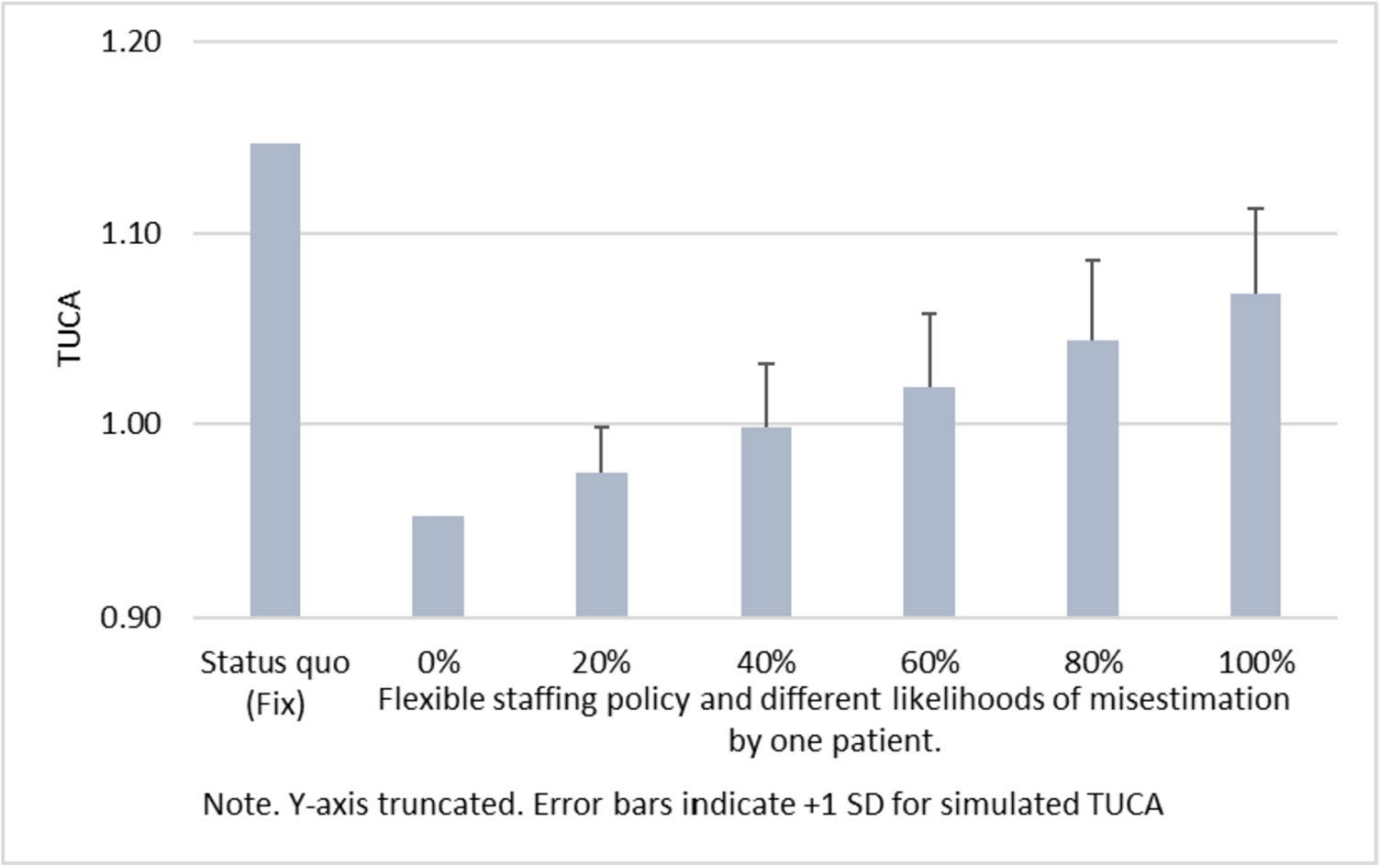
Robustness of flexible staffing policy against mis-specified number of patients

On a typical day in the NICU that we studied, 11 beds are utilized and 1.1 patients arrived/departed from the unit per day (i.e. 0.37 patients per shift). We thus cannot rule out that the realized patient volume differs from the expected one, but it is quite unlikely that the patient volume differs by more than one patient. We now consider different scenarios in which the expected number of patients differs from the real number of patients. With probability *p* the actual number of patients is mis-specified by one patient, that is, either one patient higher or one patient lower than the planned number of patients with a probability of *p/2* each. Staffing levels are determined based on the expected number of patients and Fig. 3 shows the expected TUCA for flexible staffing. We see that TUCA increases with the probability of misestimation by one patient but the figure also indicates that the flexible staffing policy outperforms the fixed staffing rule, even in the scenario in which the planned patient volume is off by one patient all of the time.

### Mis-specified patient severity

**Fig. 4:**
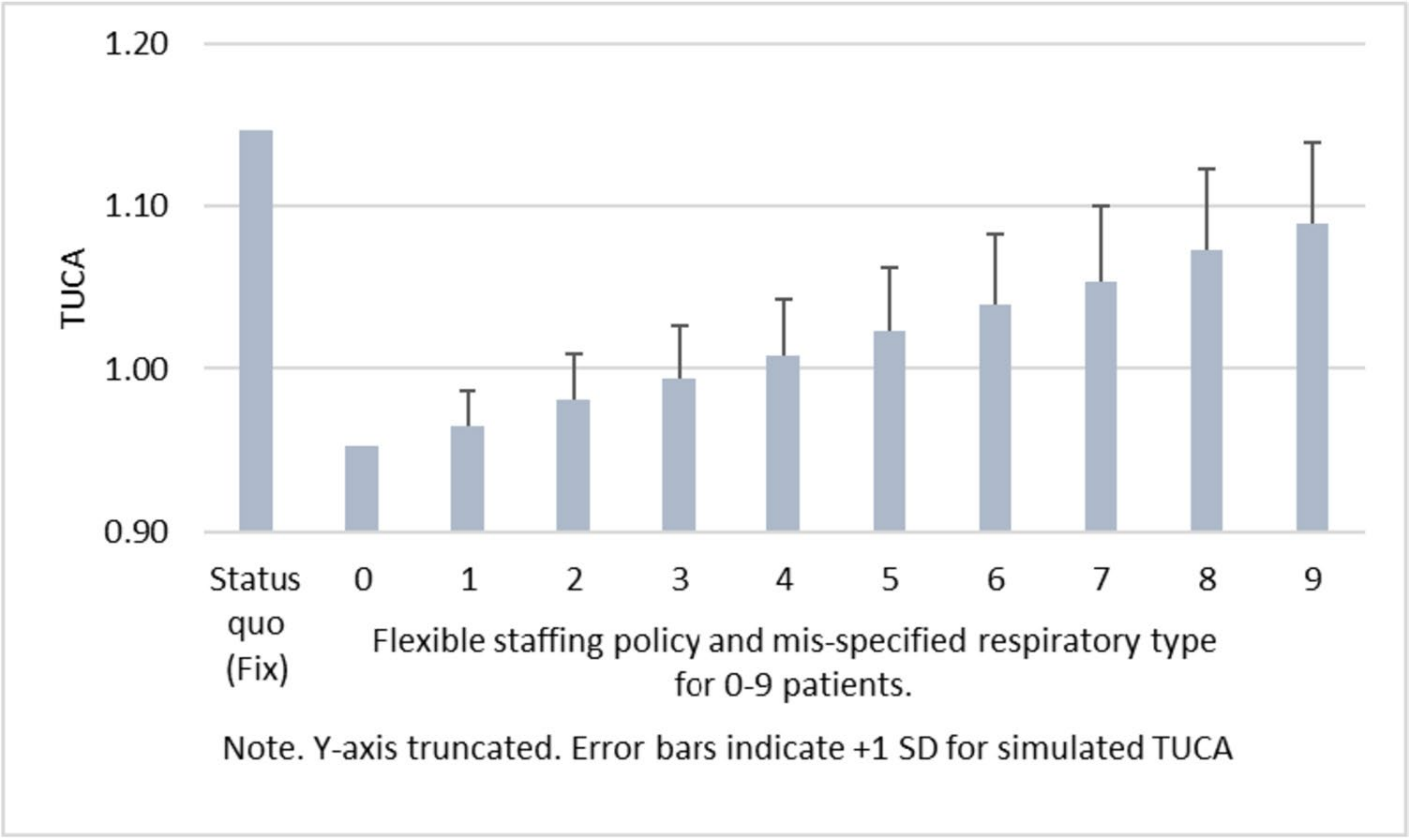
Robustness of flexible staffing policy against mis-specified patient severity

Another reason why the realized patient mix can deviate from the expected one is due to wrongly assessed respiratory types. The scenarios presented in Fig. 4 outline the consequences of having misestimated the respiratory type of 1–9 patients. In the simulation we randomly choose 1–9 patients to be potentially mis-specified. The distribution of the random draw follows the empirical distribution of respiratory types observed in our data set. Again, we see that expected TUCA increases with the number of patients whose respiratory types was estimated incorrectly. However, the flexible staffing policy still outperforms the fixed staffing rule, even when the respiratory types turn out to be potentially different for a substantial number of patients.

### Mis-specified number of patients and patient severity

**Fig. 5:**
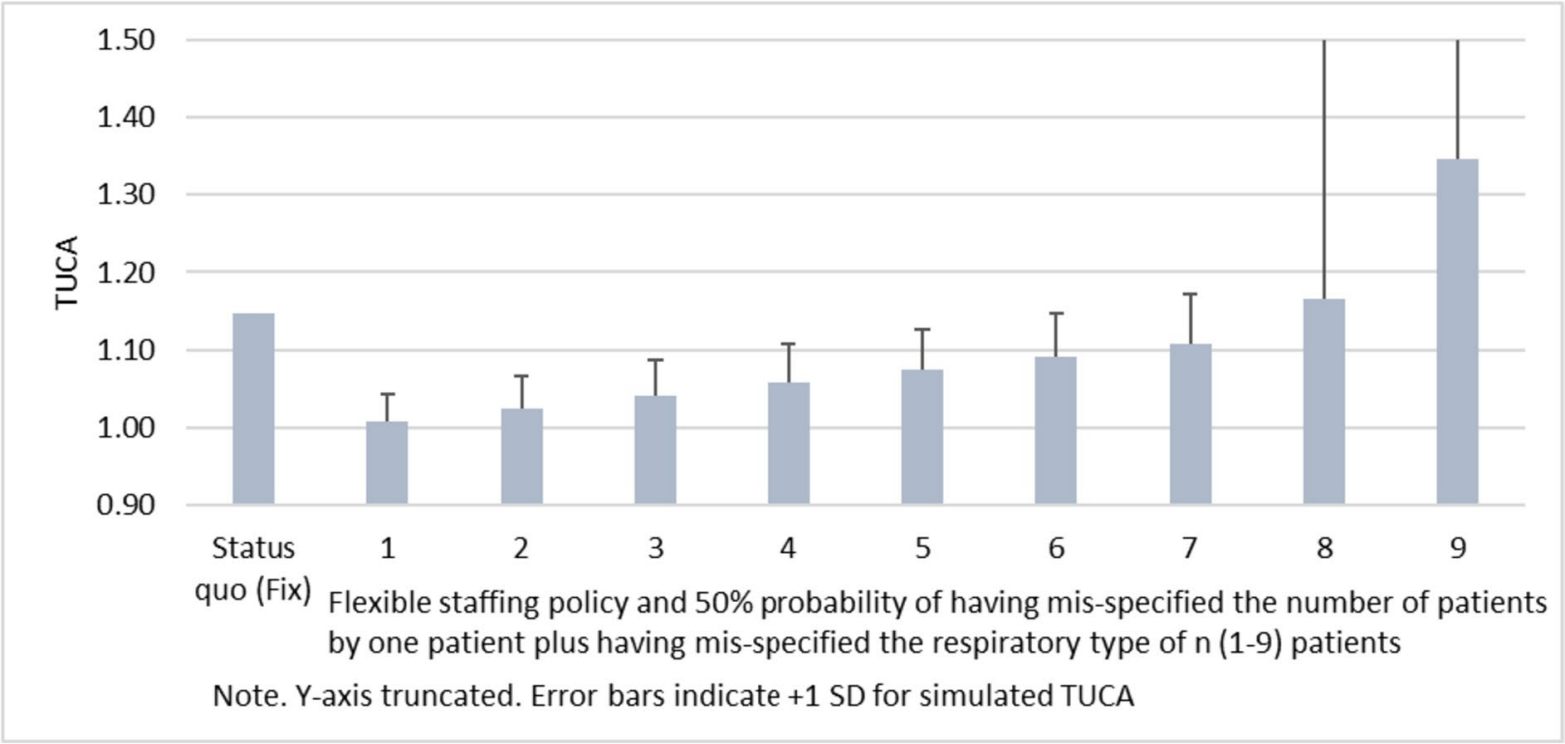
Robustness of flexible staffing policy against mis-specified number of patients and patient severity

In this section we explore the boundary conditions of the approach by simulating TUCA for scenarios that combine both types of deviations from planned to realized patient mix. Each scenario is characterized by two features: (i) a 50% probability of having mis-specified the number of patients by one patient (either one patient higher or one patient lower), and (ii) having mis-specified the respiratory type of n patients, with *n* = 1 (2,…,9) in case of scenario 1 (2,…,9). Figure 5 shows the expected TUCA for these different scenarios and indicates that minor mis-specifications do not seem to cause severe implications on TUCA. However, we find that a mis-specification of eight or more patients combined with an under- or overestimation of the number of patients leads to a TUCA that exceeds the TUCA of the fixed staffing regime. Thus, in cases with very low information on the patient mix, the fixed staffing system is more robust than the flexible staffing policy.

## Discussion and conclusion

This case study features efficient nurse allocation and improved staffing decisions for healthcare providers. With providers struggling to fill nurse vacancies and staff effectively, it is of growing importance that they identify and exploit situations where staffing an additional nurse generates the maximum benefit. We claim that, in the critical care context, the reduction of expected waiting *times until care arrives* (TUCA) for patients serves as a valid indicator for assessing the benefit of staffing an additional nurse. Based on that reasoning, we set up a queuing model that treats demand as a stochastic process that mitigates the shortcomings of mere average workload statistics. This approach allows us to determine TUCA for different staffing scenarios to then identify situations where nurses yield the most benefit. The queuing model is based on a granular level of care-events to adequately reflect the care intensity variation *among* patients and *within* patients over time. Parametrizing the queuing model with real data allows us to quantify the implications of different staffing levels, as well as to map efficiency frontiers for a range of staffing scenarios that illustrate the trade-off between better care and lower direct nursing costs.

We argue that TUCA can serve as a proxy for process quality and that flexible staffing scenarios may well improve care effectiveness. From an operations management perspective, it might not be surprising that a flexible versus fixed staffing level leads to a better quality of care-costs trade-off. From a clinical perspective, though, this is essential since a flexible staffing policy especially averts ultra-high TUCA values that compromise patient safety. A flexible staffing policy would thus level nursing workload and thereby reduce the risk that essential care practices are omitted [45, 46].

No research is free of limitations and the results should be interpreted this light. Within the queuing model, we assume a full pooling of care events; that is, no dedicated nurse(s) will handle a given care event. In clinical practice, nurses are often assigned to individual patients at shift onset. While nurses may assist other patients when required – their own workloads permitting – they mainly provide care for their assigned patients. Obviously, assuming inflexibly dedicated nurses would lead to huge increases in time until care arrives, while full pooling clearly rules out continuity of care. Detailed discussion of this trade-off is beyond the scope of our study, but it does offer an interesting avenue for future research.

We further acknowledge a lack of distinction within a group of care events. Naturally, this limitation in the queuing model reflects a critical care setting characterized by incidents that demand immediate response by health care professionals, and the average waiting times we report should be interpreted subject to this issue. While prioritization of critical care events keeps the waiting times for critical incidents, such as respiratory arrest, at lower levels than the average TUCA, noncritical incidents will consequently incur extended waiting times. To a certain extent, a rise in TUCA should, therefore, not automatically presume serious detrimental effects. Still, quality of care suffers when long wait times often delay care for non-critical events.

The case study illustrated the potential of using queuing theory to inform staffing decisions and the question instantly arising is how to actually implement the approach. The queuing model assumes that the number of patients and patient mix are known *prior* to the shift, thus providing the required data to assess marginal benefits of staffing another nurse while permitting nurse capacity adjustment. Therefore, a fundamental prerequisite for successful implementation is a reliable forecast of expected demand. Relying on demand forecast, however, means that we cannot level demand surges at all times. But with adequate forecasts we will be able to reduce the risk of excessive workload situations. Real-time data on patient mix is therefore a must for successful implementation. For hospitals using IT systems that collect (daily) patient-level data electronically with the ability to implement information systems specifically for intensive care units [47], the technical prerequisites appear in place. However, which patient data must be integrated and how frequently the model must be updated to yield adequate forecasts remain a fruitful avenue in future practice-oriented research, bearing in mind that new information technology might lead to additional staffing requirements [48]. Having data on care-event level in large quantities also allows for more granular queuing models, and for challenging our assumptions of Markov arrivals and stationarity, and potentially for developing more suitable models.

The approach we describe in the paper requires a threshold that states until which reduction in TUCA an additional nurse should be staffed. This threshold symbolizes the required value of an additional nurse: how many minutes of TUCA reduction do we want to achieve in order to invest in an additional nurse? Thus, this threshold should be derived from the hospital’s aspired service level, which basically describes how much the hospital is willing to pay for a unit reduction in TUCA. The literature is rich with suggestions how to measure willingness-to-pay [e.g., 49, 50]. But these approaches come with strengths and weaknesses that decision-makers need to assess given their organizational context and the question whether such a threshold is determined top-down as opposed to having affected professionals participating in setting the threshold. An alternative approach to defining a threshold value is to define a maximum level of TUCA for all situations and to staff nurses until this level is reached. Finally, one could adjust the approach by using different objective functions such as, e.g., minimizing the range in waiting times [51].

Assessing the marginal benefits of one more nurse and staffing effectively calls for some flexibility in aligning staffing levels with anticipated care demand. Having highly specialized nurses on call to be staffed on short notice (or releasing nurses on short notice) may incur additional costs that detract from achieved benefits. A related issue for implementation is whether such flexible staffing regimes are acceptable to nurses and align with human resource policy. From an economic perspective, we might argue that flexible policies win nurses favor when their flexibility is rewarded in their compensation [52] (one approach that has been proposed by Fortin and Douglas (2006) is bidding for additional shifts [53]). However, extensive consultations with clinicians and practitioners from related organizations indicated that the willingness to take on extra shifts seems to be decreasing despite considerable higher tariffs paid for extra shifts. Higher tariffs do not necessarily imply a higher commitment to take on extra shifts, nurses seem to favor a reduction in working hours instead. Our consultations also revealed mixed experiences with implementing on-call schemes that are not undisputed among nurses. Some organizations fail to implement such schemes due to severe pushback, whereas other organizations succeed to overcome opposition. These observations underline the importance of a thorough implementation strategy and the role of leadership when communicating and implementing strategic changes such as on-call schemes [54].

Another attempt to increase flexibility in staffing is through setting up float pools of nurses [55]. These float pools seek to satisfy two goals. First, float pools flexibly offset staff absenteeism. Second, they enhance employer attractiveness by offering individual choice in the work schedule. Float nurses receive predictable work schedules in terms of shift times that correlate with work satisfaction [56]. Still, they are flexibly staffed in terms of location and tasks. In the event of no shift shortage, these float nurses can be assigned to alternative tasks that need regular attention after neglect in times of high workload. Such a float pool not only compensates for absenteeism but also adjusts staffing to match patient mix. This does not appear to be at odds with human resource policies, provided that float nurses have the required knowledge and education to work in these units and are highly committed to the units. In the NICU context, specific aspects are important to ensure high satisfaction and commitment—a major goal of nurses is being able to primarily focus on the needs of patients and parents [57]. Whether organizations can set up float pools effectively is likely contingent on the organizational context. Larger organizations, for instance, may find it easier to enlist a flexible staffing regime with a float pool of nurses that exploits economies of scale and pooling benefits. Organizations then need to decide strategically how many nurses to hire for each unit and for the float pool, how to translate nurse preferences into rosters, and how to allocate pool nurses to the units [58]. The queuing application outlined in this paper could be informative for the latter but might require adjustments if transferred to different contexts. Different wards/ settings probably have different key indicators that trigger care events, which need to be identified and estimated to parametrize the model. Whether one requires manual data collection for this or whether alternative approaches (e.g. webcams, tracking devices, etc.) might be suitable for data collection depends on the setting and needs to be assessed case by case.

The case study presented in this paper thus highlights the potential and scientific merits of using queuing theory to improve staffing decisions while simultaneously identifying the practical challenges. Therefore, our long-term goal is to design an implementation study that transfers our learning into practice to evaluate the approach empirically. Implementing the approach in practice means that we intend to change staffing policies – with direct implications for the patient care process. Such an intervention thus requires a different study protocol and approval process than those for the observational study. Logically, the implementation step must follow separately.

## Supplementary Information

Below is the link to the electronic supplementary material.

Supplementary file1 (PDF 173 kb)

## Data Availability

The data that support the findings of this study are not openly available due to privacy reasons and are available from the corresponding author upon reasonable request.
