# Supplementary material for: The potential of patient-based nurse staffing – a queuing theory application in the neonatal intensive care setting
Source: Health Care Manag Sci. 2024 Jan 30;27(2):239–53. doi: 10.1007/s10729-024-09665-8 (PMC11637038; doi:10.1007/s10729-024-09665-8)
Supplement: Supplementary file 1 — Supplementary file1 (PDF 173 kb) [file 10729_2024_9665_MOESM1_ESM.pdf]

The potential of patient-based nurse staffing –

a queuing theory application in the neonatal intensive care setting

APPENDIX

This appendix accompanies the paper "The potential of patient-based nurse staffing – a queuing theory application in the neonatal intensive care setting". It elaborates on the technical details of the paper and contains additional analyses.

Table of content

A1. Data Collection Protocol 2

A2. Deriving input parameters for the queuing models 3

A2.1 Why differentiation between shifts is reasonable 3

A2.2 Why differentiation between types of respiratory support is reasonable 4

A3. Model input validity 5

References 9

A1. Data Collection Protocol

The taxonomy of nursing tasks recorded is provided in Table A1-1.

Table A1-1: Taxonomy of nursing tasks recorded

Category	Nursing tasks recorded
**Direct Care**	- Observation/assessment, alarms, documentation, charts, seeking medical advice, liaising with physicians (directly patient-related)<br>- Patient admission/discharge (non-administrative), assisting in delivery suite, transporting babies, changing incubator<br>- Weighing, bathing, cleaning, caring for nappies, skin care, positioning, oral care, caring for endotracheal tube, oral/endotracheal suctioning, changing of naso-gastric tubes, caring for gastrostomy tubes, changing of stoma bags, wound care, rectal irrigation<br>- Assisting with: blood sampling (iv, ia), endotracheal intubation, resuscitation, insertion of chest/peritoneal drains, peripheral venous lines, central venous catheters, arterial lines, X-rays, ultrasound, councils (e.g. surgeons, ophthalmologists)<br>- Urinary bladder catheterization, preparation of blood exchange transfusion system, taking capillary blood samples, blood gases and analysis of blood glucose/electrolytes/bilirubin, performing phototherapy, sampling for microbiology (skin-, nasal-, rectal swabs, tracheal fluids)<br>- Administration of drugs (oral, iv route), changing iv and ia fluids<br>- Nutrition: feeding, orally, via naso-gastric tube<br>- Developmentally supportive care, skin-to-skin care contact, assisting with physiotherapy, performing passive physiotherapy, multi-sensory stimulation, instructing parents
**Indirect care**	- Admission and discharge planning, liaising with other units for transfer planning<br>- Making up breast milk feeds, fortifiers<br>- Cleaning equipment, checking emergency tools, arranging medical tests, general hygienic measures (e.g. washing and disinfecting hands)<br>- Answering the phone, interacting with parents, dealing with bereaved parents, dealing with visitors, shift handover, ward rounds, supervision of ward

	- Prenatal patient visits/psycho-social meetings, discussion with multi-disciplinary team, liaising with physicians (not directly patient-related)
**Administration**	- Documentation (general, no patient-related), practical instructions (equipment updates), supplies<br>- General management, sorting off duty/staffing, ward meetings, liaising with psychologists<br>- Individual performance review/ interviews, social issues
**Other**	-Staff personal needs, staff break, meal break, off ward (external meetings/conferences)<br>- Organizational tasks, housekeeping tasks, giving/receiving instructions<br>- Assisting in clinical studies/research, personal studies, taking care of medical students

A2. Deriving input parameters for the queuing models

A2.1 Why differentiation between shifts is reasonable

We argue in the main paper that structural features, such as the shift types (early, late, and night) inherent in current rosters, are likely to affect care demand because the shifts differ with respect to the procedures occurring in them. Planned procedures, for instance, exchanging the respiratory support material, sonography, taking blood samples, and scheduled C-sections, occur more often in early shifts. In addition, day shifts might be characterized by higher acoustic levels due to, for instance, the presence of more health practitioners and visitors on the ward. Infants might respond to this physiologically and behaviorally, thus triggering care events [1]. Our data pattern allows us to determine the time each nurse spends on providing direct care, and we aggregate this across all nurses. Please note that while this metric may be interpreted as the execution by the nurse of direct care, the remaining time will be filled with indirect care and administrative and other activities. Our empirical analysis indeed indicate that the shift types differ in the proportion of time spent on providing direct care; early shifts tend to require more nursing time on direct care (66.6%) than late (57.3%) and night shifts (48.1%). The Kruskal-Wallis test also

rejects the equality of distributions ($p<0.01$). This result supports the approach of analyzing the shifts separately. Note that the fraction of direct care per nurse is higher in early shifts even though more nurses are available; therefore, there appears to be much higher care demand in early shifts compared to late and night shifts. Based on this observation, we will not only differentiate situations based on the patient mix, but also on the shift type in the paper.

A2.2 Why differentiation between types of respiratory support is reasonable

To account for differences between patients, we require a patient indicator that is easily observable ex ante and that discriminates well between different levels of care intensity. We have argued in the paper that it is reasonable to focus on the type of respiratory support. We now show empirically that the patients' care demand indeed differs between the four types considered ((1) mechanical ventilation, (2) nCPAP, (3) high-flow cannula, and (4) a miscellaneous category containing all infants without respiratory support).

For each type of respiratory support, we determine how much of the available nurse capacity an infant requires for direct care. Note that our data is collected at nurse-level and we are not observing all nurses (or all patients) simultaneously. Take for instance the following example: Four nurses provide care for ten infants in the complete NICU, with five of the infants receiving nCPAP. While four nurses are actually providing care, we only observe three nurses during the observation period. Nurse A spends 60% of her time on nCPAP infants, Nurse B 40%, and Nurse C 20%; that is, the average observed care demand per nurse and nCPAP respiratory support type is 40%. With four nurses on duty, the cumulated care demand for nCPAP infants in the complete NICU is 160% of an average nurse capacity (40% * 4 nurses on duty). With five infants receiving nCPAP, an individual infant thus requires on average 160%/5 = 32% nurse capacity. We proceed similarly for all respiratory support types, i.e. we multiply the average observed care demand per nurse for each type of respiratory support by the number of nurses on duty to obtain the

total care demand per type of respiratory support in the complete NICU. In the second step, this metric is divided by the number of treated patients of this respiratory support type, resulting in the nursing workload per patient for this type (measured in nurse capacity). Repeating the above for every observation day in our sample provides us with a distribution of care demand per respiratory support type. Notably, the Kruskal-Wallis tests reject the equality of distributions ($p<0.05$) indicating that the type of respiratory support indeed correlates with varying levels of care demand. This finding is in line with a medical study that used a high-risk patient subset of the data and found significant differences in nursing workload depending on the respiratory support type [2].

A3. Model input validity

One of the assumptions that we make is that the arrivals of care events follow a Poisson process. This is a convenient assumption from a modeling perspective as Poisson processes rely on a single parameter that we can estimate using our data. In health care, unscheduled patient arrivals to service systems, such as intensive care units and emergency departments, are indeed in line with Poisson processes [3-5]. When modeling the entire unit, i.e. care events across all patients, the assumption of a Poisson process is fairly reasonable because we are facing a neonatal intensive care setting that is characterized by stochastic incidents: Infants might generate alarms due to stochastic fluctuations of heart rates and oxygen saturation, spontaneous movements of infants might dislocate tubes/ nasal masks etc. Therefore, it appears reasonable to assume that care events arise one at a time, are expected to occur independently of how many other care events occurred across all patients, and that care events are independent of a given moment in time during a particular shift. We also analyze the care events in our data set and find that the distribution of observed starts of care events appears to be in line with a Poisson distribution (see Figure A3-1).

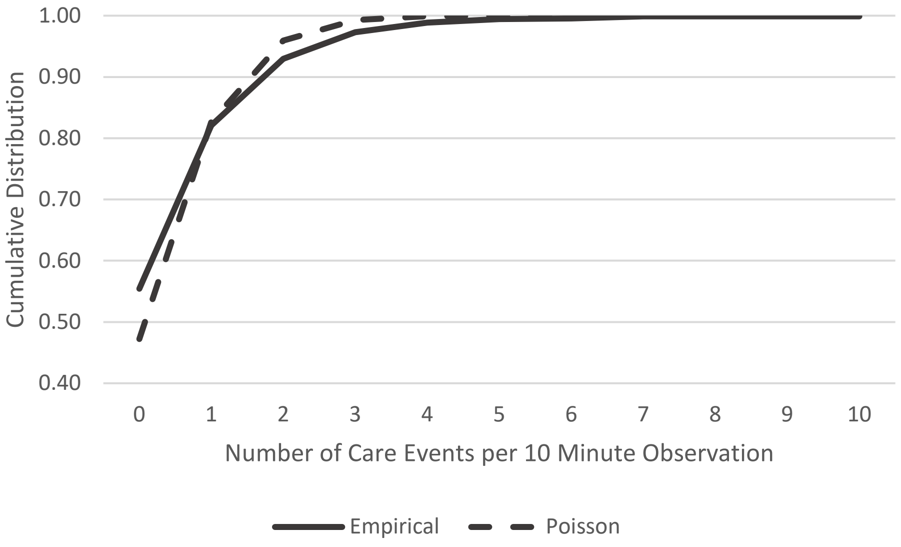

Figure A3-1: Comparison empirical and theoretical cumulative distributions (all patient types)

The second assumption of the Markovian model is that the duration of service time for the patient mix is exponentially distributed. This assumption is consistent with relevant literature on ICU modeling [6] and stems from the fact that most care events are quick check-ups requiring very little time, while few time-consuming events, such as treatment of cardiac arrests, might occur as well. We validate this assumption for the observed patient mix using a simulation model. In simulations with 1,000 runs each, we model the duration as an aggregate of resampled combinations of the left-censored, right-censored and both-sided-censored scenarios and compute the coefficient of variation (SD(x)/E(x)), which is 1.00 for exponentially distributed variables. Using our empirical data, the simulation results report an average coefficient of variation of 0.97 (between 0.90 and 1.04) for early shifts, 1.07 (between 1.00 and 1.34) for late shifts, and 1.26 (between 1.15 and 1.39) for night shifts. We illustrate the comparison of observed, that is simulated, and theoretical cumulative distributions of durations for one simulation run of an early, late and night shift each in Figure A3-2 - Figure A3-4. We conclude that particularly for early and late shifts, the assumption of care event durations following an exponential distribution appears to be reasonable. For

night shifts, we rely on approximations for distributions with coefficients of variation greater than 1.00 based on the Kingman equation [7].

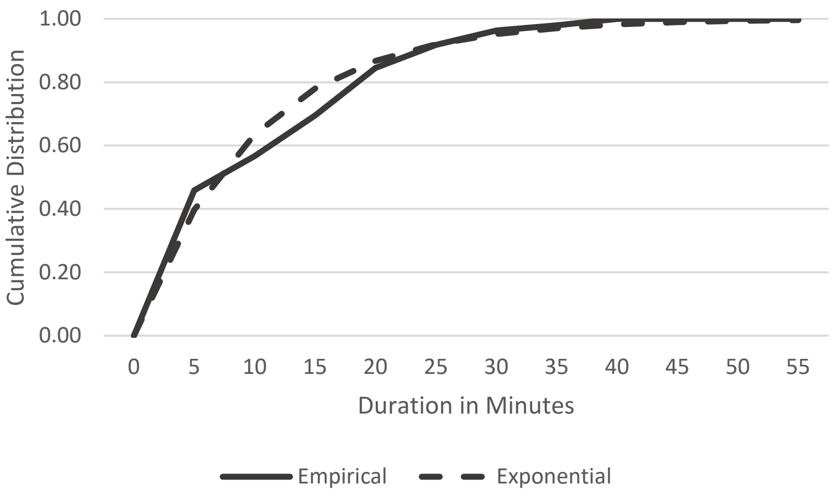

Figure A3-2: Comparison empirical and theoretical cumulative distributions for one simulation instance (early shift)

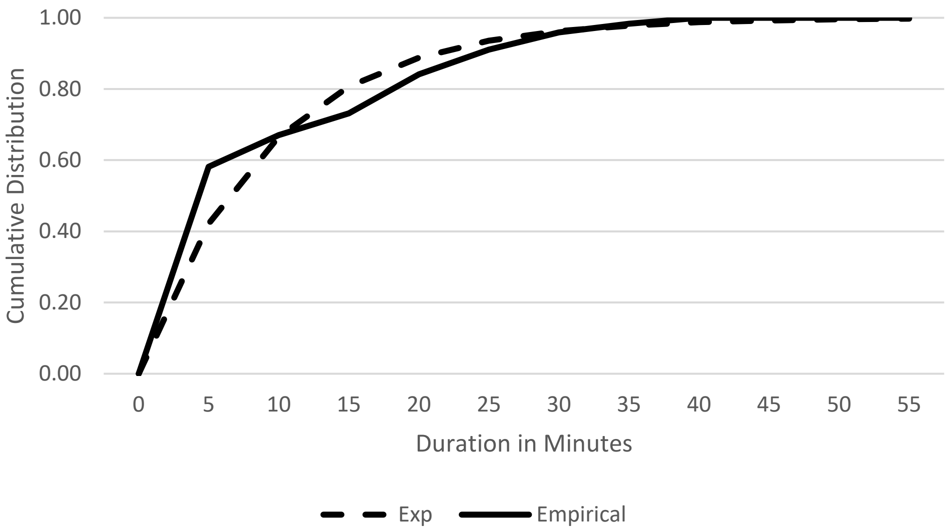

Figure A3-3: Comparison empirical and theoretical cumulative distributions for one simulation instance (late shift)

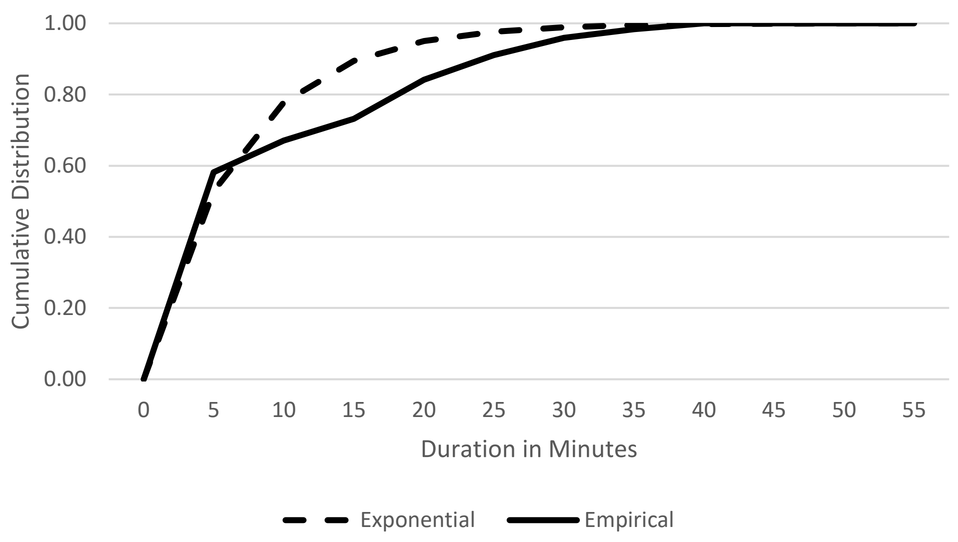

Figure A3-4: Comparison empirical and theoretical cumulative distributions for one simulation instance (night shift)

Using the aforementioned distributional assumptions on arrival processes and service times, with their underlying parameters derived empirically, we finally assess whether our queuing model predicts proportional values of time spent on providing direct care across all nurses that are in line with the proportional values that we observe in our data, i.e., the values reported in Section A2.1. Relating the queuing model's predicted arrival and duration of care events for the patient mix of our observation period to the NICU's available nursing time, our model predicts that in early shifts, 66.3% of the time is spent on direct care (observed: 66.6%); in late shifts, the model predicts 53.3% (observed: 57.3%); and for night shifts, the prediction is 48.7% (observed: 48.1%). These results show that our queuing model and our empirical data are consistently aligned.

References

[1] Zahr, L. K., Balian, S., 1995. Responses of premature infants to routine nursing interventions and noise in the NICU. Nursing Research, 44(3), 179-185.

[2] Langhammer, K., Sülz, S., Becker-Peth, M., & Roth, B. (2017). Observational study shows that nurses spend more time caring for mechanically ventilated preterm infants than those receiving noninvasive ventilation. *Acta Paediatrica*, *106*(11), 1787-1792.

[3] Green, L.V., Soares, J., Giulio, J., & Green, R., 2006a. Using queueing theory to increase the effectiveness of physician staffing in the emergency department. Academic Emergency Medicine, 13(1), 61-68.

[4] Green, L. 2006b. Queueing analysis in healthcare. In: Hall, R. (Ed), Patient flow: reducing delay in healthcare delivery, 281-307, Springer US, New York.

[5] Kim, S.C., Horowitz, I., Young, K.K., Buckley, T.A., 1999. Analysis of capacity management of the intensive care unit in a hospital. European Journal of Operational Research, 115(1), 36-46.

[6] Litvak, N., Van Rijsbergen, M., Boucherie, R.J., van Houdenhoven, M., 2008. Managing the overflow of intensive care patients. European Journal of Operational Research, 185(3), 998-1010.

[7] Curry, G.L., Feldman, R.M., 2010. Manufacturing systems modeling and analysis. Springer Science & Business Media.
